# Neurofilament heavy chain in secondary progressive multiple sclerosis

**DOI:** 10.1177/13524585241311212

**Published:** 2025-01-22

**Authors:** Floriana De Angelis, Francesca Ammoscato, Richard A Parker, Domenico Plantone, Anisha Doshi, Nevin A John, Thomas Williams, Jonathan Stutters, Dave MacManus, Klaus Schmierer, Frederik Barkhof, Christopher J Weir, Gavin Giovannoni, Jeremy Chataway, Sharmilee Gnanapavan

**Affiliations:** Queen Square Multiple Sclerosis Centre, Department of Neuroinflammation, University College London Queen Square Institute of Neurology, Faculty of Brain Sciences, University College London, London, UK; National Institute for Health and Care Research, University College London Hospitals Biomedical Research Centre, London, UK; Blizard Institute, Barts and The London, London, UK; Edinburgh Clinical Trials Unit, Usher Institute, University of Edinburgh, Edinburgh, UK; Queen Square Multiple Sclerosis Centre, Department of Neuroinflammation, University College London Queen Square Institute of Neurology, Faculty of Brain Sciences, University College London, London, UK; Department of Medicine, Surgery and Neuroscience, University of Siena, Siena, Italy; Queen Square Multiple Sclerosis Centre, Department of Neuroinflammation, University College London Queen Square Institute of Neurology, Faculty of Brain Sciences, University College London, London, UK; Queen Square Multiple Sclerosis Centre, Department of Neuroinflammation, University College London Queen Square Institute of Neurology, Faculty of Brain Sciences, University College London, London, UK; Department of Medicine, School of Clinical Sciences, Monash University, Melbourne, VIC, Australia; Department of Neurology, Monash Health, Melbourne, VIC, Australia; Queen Square Multiple Sclerosis Centre, Department of Neuroinflammation, University College London Queen Square Institute of Neurology, Faculty of Brain Sciences, University College London, London, UK; Queen Square Multiple Sclerosis Centre, Department of Neuroinflammation, University College London Queen Square Institute of Neurology, Faculty of Brain Sciences, University College London, London, UK; Queen Square Multiple Sclerosis Centre, Department of Neuroinflammation, University College London Queen Square Institute of Neurology, Faculty of Brain Sciences, University College London, London, UK; Blizard Institute, Barts and The London, London, UK; Queen Square Multiple Sclerosis Centre, Department of Neuroinflammation, University College London Queen Square Institute of Neurology, Faculty of Brain Sciences, University College London, London, UK; National Institute for Health and Care Research, University College London Hospitals Biomedical Research Centre, London, UK; Department of Radiology and Nuclear Medicine, VU University Medical Centre, Amsterdam, The Netherlands; Department of Medical Physics and Biomedical Engineering, Centre for Medical Image Computing (CMIC), University College London, London, UK; Edinburgh Clinical Trials Unit, Usher Institute, University of Edinburgh, Edinburgh, UK; Blizard Institute, Barts and The London, London, UK; Queen Square Multiple Sclerosis Centre, Department of Neuroinflammation, University College London Queen Square Institute of Neurology, Faculty of Brain Sciences, University College London, London, UK; National Institute for Health and Care Research, University College London Hospitals Biomedical Research Centre, London, UK; Blizard Institute, Barts and The London, London, UK

**Keywords:** Neurofilaments, progressive multiple sclerosis, biomarkers

## Abstract

**Background:**

Biomarkers are needed to track progression in MS trials. Neurofilament heavy chain (NfH) has been underutilized due to assay limitations.

**Objective:**

To investigate the added value of cerebrospinal fluid (CSF) NfH in secondary progressive multiple sclerosis (SPMS) using contemporary immunoassays.

**Methods:**

This exploratory study was part of the MS-SMART trial. Clinical assessments (including expanded disability status scale, upper and lower limb function, visual acuity and symbol digit modalities test (SDMT)), CSF and serum sampling were acquired at baseline (*n* = 54), 48 and 96 weeks. Brain magnetic resonance imagings (MRIs) were obtained at baseline and 96 weeks. The NfL and NfH were measured using single-molecule array assay.

**Results:**

Baseline CSF NfH and NfL correlated with information processing speed at 96 weeks, with CSF NfH showing stronger correlations (*r* = −0.49 for SDMT) than CSF NfL (*r* = −0.37 for SDMT). Baseline CSF NfL predicted poorer hand dexterity at baseline, 48 and 96 weeks. CSF NfH was the only predictor of cortical grey matter at baseline, while baseline CSF NfL was the only predictor of brain atrophy at 96 weeks. Serum neurofilaments showed limited associations.

**Conclusion:**

CSF neurofilaments are better outcomes than serum neurofilaments in small SPMS studies. CSF NfH and NfL variably predict worsening hand function, information processing speed and brain volume loss, possibly reflecting complementary aspects of neurodegeneration.

## Introduction

Multiple sclerosis (MS) is a chronic, immune-mediated disease of the central nervous system (CNS) characterized by inflammation, myelin and axonal injury.^1^ Axonal loss is a key pathological feature underpinning chronic disease deterioration, although synaptic damage and loss contribute.^2^ Axonal loss occurs throughout MS, mainly through two mechanisms: first, as a result of axonal transection in acutely inflamed focal lesions;^1^ and second, as a delayed degeneration from earlier damage when compensatory mechanisms fail,^3^ which is a major cause of chronic disability accrual.^4^ Outcome measures to assess neuroprotective agents’ efficacy in the setting of delayed axonal loss in MS are limited.^5,6^ Clinical trial measures are insensitive to detecting early worsening and require large sample sizes.^7^ Thus, reliable surrogate markers of axonal damage and progression remain an unmet need.

Neurofilaments represent the main neuronal structural proteins, mostly found in axons composed of light (NfL), medium and heavy (NfH) chain subunits.^8^ Pathological processes inducing neuroaxonal damage like MS^9^ release neurofilaments into cerebrospinal fluid (CSF), and, depending on damage extent, into peripheral blood. Due to their abundance and neuron specificity, neurofilaments show promise as injury markers. In relapsing-remitting MS, NfL is associated with clinical and magnetic resonance imaging (MRI) inflammatory activity and predicts disability accrual.^10–15^ CSF NfL levels increase during relapses, correlating with disability and MRI lesion load.^16,17^ However, in progressive MS, disease activity is modest, while neurodegeneration is relentless. There are differences in how NfL and NfH are released into the CSF. NfL diffuses quickly due to lower molecular weight and phosphorylation status, with consequent rapid degradation, whereas NfH degrades slowly.^8^ In addition, NfH is mostly located in large, myelinated axons and is more likely to be released during the later stages of MS.^17^ NfH, therefore, becomes the predominant axonal protein in CSF in progressive MS, while NfL marks early axonal injury. Considering this pathological aspect of progressive MS, NfH may serve as a better outcome than NfL. Indeed, CSF NfH increases during irreversible late-stage axonal degeneration of progressive MS.^14,16,18,19^

In such a scenario, testing a trial design based on CSF neurofilament levels as a readout for axonal damage and neuroprotection is reasonable. However, until recently, NfH could not be accurately measured due to technical limitations. Therefore, its role in progressive MS has rarely been investigated in contemporary research studies.

The Multiple Sclerosis-Secondary Progressive Multi-Arm Randomization Trial (MS-SMART; NCT01910259) was a multi-arm, placebo-controlled trial investigating the neuroprotective role of amiloride, fluoxetine and riluzole in people with secondary progressive multiple sclerosis (SPMS).^20–22^ The trial was completed successfully; however, no evidence of a treatment effect was detected for any of the three drugs tested on the primary outcome. Several optional substudies were undertaken within the main MS-SMART trial framework, which included a CSF substudy. While the pre-planned primary CSF substudy analysis was negative, showing no difference in CSF NfL and NfH between any treatment arms,^22^ this current exploratory analysis aims to look at CSF NfH measured with a contemporary immunoassay,^23,24^ as a predictor of disability, brain atrophy and MRI disease activity in a cohort of people with SPMS. The hypothesis behind our study is that CSF NfH reflects neurodegeneration better than CSF NfL in people with progressive MS. Therefore, the main aim of our study was to specifically investigate CSF NfH as a predictor of disease severity and worsening. As secondary outcomes, we also looked at serum neurofilament light and heavy chains.

## Materials and methods

### Participants

The present study was a substudy embedded in the MS-SMART trial. Participating subjects at the University College London (UCL) site were invited to participate in this optional CSF substudy. The main eligibility criteria, as per the MS-SMART trial, were: age 25–65 years included, Expanded Disability Status Scale (EDSS) score 4.0–6.5 and diagnosis of SPMS characterized by gradual worsening due to disease progression rather than relapses as a major cause of increasing disability in the preceding 2 years. Progression could be established from either an increase of at least one point in EDSS or clinical documentation of increasing disability in medical records in the preceding 2 years. Patients on disease-modifying therapies or treated with steroids within 3 months of their baseline visit were excluded.^20–22^ The MS-SMART study and its substudies were approved by the local Ethical Committee, and written consent was obtained from all participants.

### Procedures and outcomes

Participants underwent lumbar puncture (LP) at baseline, 48 and 96 weeks. We did not systematically use an atraumatic needle for LP. CSF and paired serum samples were collected at each time point. NfL and NfH were measured in both CSF and serum. In addition, participants underwent neurological examination inclusive of EDSS, timed 25-foot walk (T25FW), nine-hole peg test (9HPT), Paced Auditory Serial Addition Test (PASAT), Symbol Digit Modalities Test (SDMT) at baseline, 48 and 96 weeks. Brain MRI was performed for all subjects at baseline, 24 and 96 weeks. MRI-derived measures included percentage brain volume change (PBVC), T2 lesion volume (T2LV), the number of new or enlarging T2 lesions, whole brain volume (WBV) and grey matter volumes (deep grey matter volume [DGMV] and cortical grey matter volume [CGMV]).

### MRI analysis

We obtained the following scans: 3D T1, 2D fluid-attenuated inversion recovery (FLAIR) and proton density (PD)/T2. We used Geodesic Information Flows (GIF) to extract and segment the brain and then calculated cross-sectional whole brain volumes at each MRI time point using the Structural Image Evaluation, Using Normalization, of Atrophy–Cross-Sectional (SIENA-X) and PBVC using SIENA. We manually marked T2 lesions to estimate the total T2 lesion volume. More details about the MRI analysis are described elsewhere.^20–22^

### CSF and serum neurofilament analysis

CSF and paired serum specimens were collected, processed, aliquoted and stored at −80°C according to a standardized operating procedure. CSF and serum biomarkers were analysed at the Blizard Institute of Queen Mary University of London’s Faculty of Medicine & Dentistry.

Concentrations of NfL and NfH in CSF and serum were determined using the NF-Light Simoa Assay Advantage kit and pNF-Heavy Simoa Discovery kit for single-molecule array (Simoa) HD-1 analyzer (Quanterix, Billerica, MA, USA) according to instructions from the manufacturer. The calibrator (neat) and CSF samples (1:4 dilution) were loaded in duplicates. Calibrators range from 0 to 2000 pg/mL. Intra-and inter-assay variabilities were <10%.

### Statistical analysis

All statistical analyses were performed with R, version 4.4.1 (R Foundation for Statistical Computing). We compared baseline characteristics between participants in the CSF study and the entire cohort recruited at UCL using *t*-tests or Wilcoxon rank-sum tests as appropriate (Supplementary Table 2).

The statistical analyses reported in this paper were post hoc and exploratory with respect to the pre-planned MS-SMART trial statistical analysis plan. We used partial correlation analyses adjusted for age to explore associations between neurofilaments at baseline and between neurofilaments and EDSS and other clinical and MRI variables available at baseline, week 48 and week 96, and PBVC at baseline and week 96. As we could not assume normality for all the neurofilaments and some variables were not normally distributed, we used Spearman’s rank partial correlations for all the analyses. Spearman’s rank correlation is a non-parametric method that is less sensitive to not-normally distributed data and outliers. We carried out a sensitivity analysis (Supplementary Tables 2–7) after removing extreme outliers for CSF and serum NfH and NfL. A value was classified as an extreme outlier if its *z*-score was more than 3 standard deviations above or below the mean.

As neurofilaments are known to increase with age, all partial correlations were adjusted for age. Statistical significance was set at *p* < 0.05.

## Results

### Baseline characteristics

**Figure 1.**
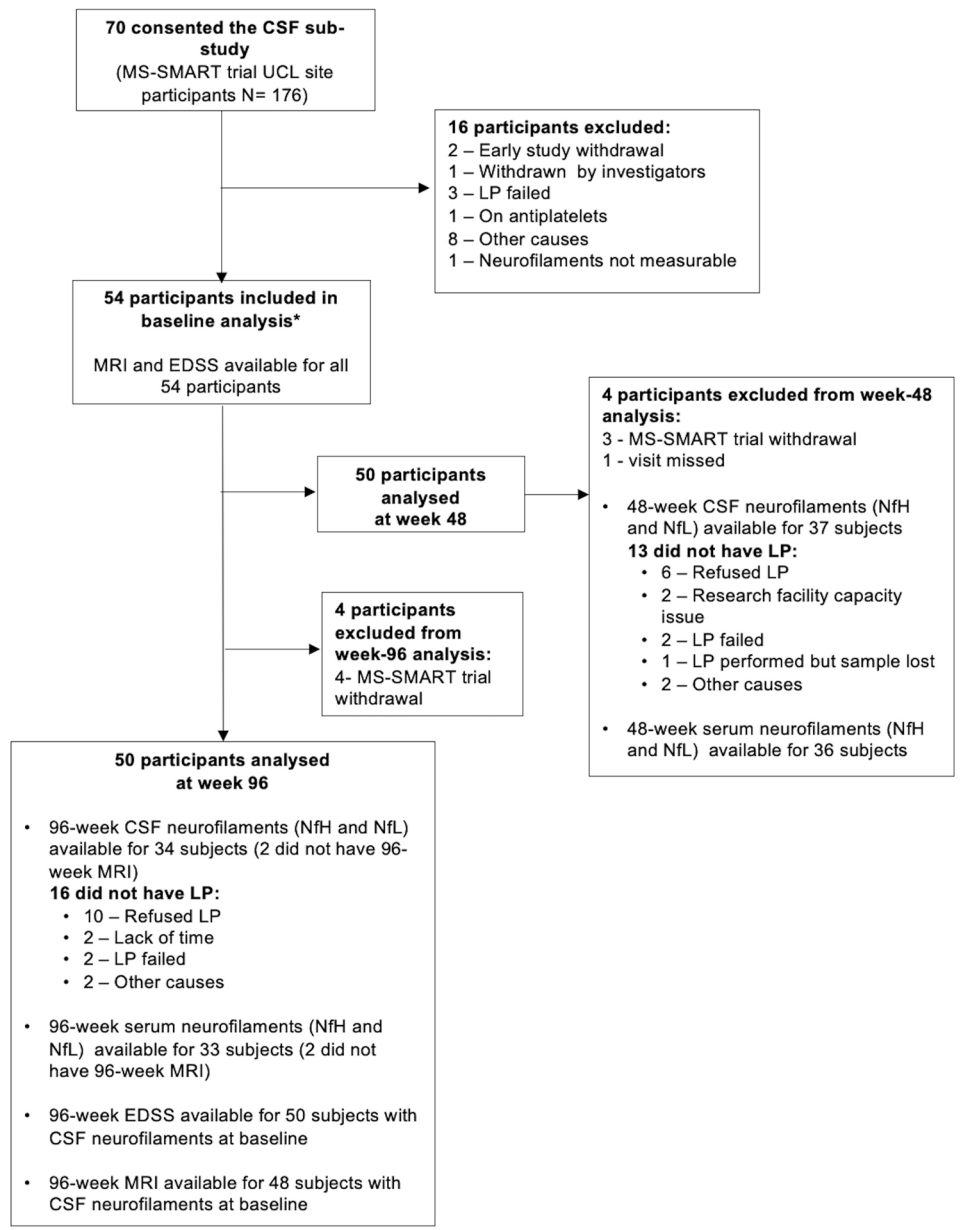
Participant disposition. CSF: cerebrospinal fluid; EDSS: Expanded Disability Status Scale; LP: lumbar puncture; MRI: magnetic resonance imaging; NfH: neurofilament heavy (chain); NfL: neurofilament light (chain); UCL: University College London.

Between December 2014 and July 2018, 55 trial participants underwent LP at baseline. We excluded one subject due to its CSF neurofilaments being not measurable or close to zero, which we interpreted as non-biologically plausible, resulting in a final baseline number of 54 subjects (Figure 1). Four subjects withdrew from the MS-SMART trial, not completing the final visit at 96 weeks. Of the remaining 50 subjects completing the trial, 37 had LP after 48 weeks, and 34 had LP at 96 weeks (Figure 1). Baseline characteristics (Table 1) reflect typical demographic, clinical and MRI features of people with SPMS, with a mean age of about 54 years, a mean disease duration of 22 years and a median EDSS of 6.0. There were no statistically significant differences in the clinical and radiological features of the patients who participated in the CSF substudy and the total number of patients participating in the MS-SMART trial at UCL (Supplementary Table 1).

**Table 1.** Baseline characteristics.

	All (*n* = 54)
Age, years	53.70 (8.16)
Female sex, %	31 (57.4)
Disease duration, years	22.05 (9.00)
Progression duration, years	8.04 (6.07)
EDSS, score	6.0 (5.625–6.5)
9HPT, seconds	40.67 (53.81)
T25FW, seconds	17.49 (27.00)
PASAT, no. of correct answers	45.5 (34.25–52.75)
SDMT, no. of correct answers	47.5 (40.25–51)
WBV, mL	1408.04 (87.92)
DGMV, mL	45.10 (4.55)
CGMV, mL	786.00 (48.81)
T2LV, mL	14.13 (11.46)
CSF NfL, pg/mL	1001.35 (511.25)
CSF NfH, pg/mL	492.18 (147.27)
Serum NfL, pg/mL	16.01 (8.95)
Serum NfH, pg/mL	94.04 (88.75)

9HPT: nine-hole peg test; EDSS: Expanded Disability Status Scale; WBV: whole brain volume; CGMV: cortical grey matter volume; CSF: cerebrospinal fluid; DGMV: deep grey matter volume; NfH: neurofilament heavy (chain); NfL: neurofilament light (chain); PASAT: paced auditory serial addition test; SDMT: symbol digit modalities test; T25FW: timed 25-foot walk; T2LV: T2 lesion volume.

Descriptive statistics are reported as percentages (number), mean (SD) or medians (interquartile range) as appropriate.

A small number of participants (*n* = 4, 7%) experienced at least one relapse in the 2 years prior to trial recruitment, and five subjects completing the trial (10%) experienced at least one relapse during the 96-week duration of the study (Supplementary Table 8).

### Baseline cross-sectional analysis: correlation between neurofilaments (NfL and NfH) in serum and in CSF

**Figure 2.**
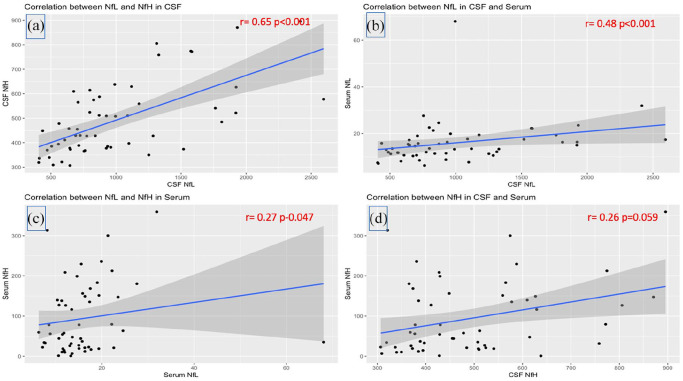
Correlation between neurofilaments in CSF and serum. Panel (a): correlation between NfL and NfH in the CSF; panel (b): correlation between NfL in CSF and serum; panel (c): correlation between NfL and NfH in serum; panel (d): correlations between NfH in CSF and serum. CSF: cerebrospinal fluid; NfL: neurofilament light (chain); NfH: neurofilament heavy (chain). Values of partial Spearman’s rank correlations adjusted for age are reported in red.

There was a significant partial correlation between NfH and NfL in CSF (*r* = 0.65, *p* < 0.001) (Figure 2(a)) and in serum (*r* = 0.27, *p* = 0.047) (Figure 2(c)). Higher CSF concentrations of NfL correlated with higher concentrations NfL in serum (*r* = 0.48, *p* < 0.001) (Figure 2(b)). However, there was no significant correlation between NfH concentrations in CSF and serum (*r* = 0.26, *p* = 0.059) (Figure 2(d)).

### Baseline cross-sectional analysis: CSF neurofilaments (NfL and NfH) are associated with cognition, upper limb function and MRI indices at baseline

At baseline, none of the neurofilaments in CSF were associated with EDSS or T25FW. Only CSF NfL, and not NfH, corresponded to a worse performance in hand function (*r* = 0.33, *p* = 0.015 vs. *r* = 0.25, *p* = 0.071). Both NfH and NfL in CSF were correlated with information processing speed as estimated by SDMT ( *r* = −0.52, *p* < 0.001 and *r* = −0.42, *p* = 0.002, respectively), but only NfL was also correlated with PASAT (*r* = −0.28, *p* = 0.040) (Table 2).

**Table 2.** Correlations between NfL and NfH with clinical and MRI variables at baseline.

Predictor	Outcome	CSF		SERUM	
		Corr. coeff.	*p*	Corr. coeff.	*p*
NfH pg/mL	EDSS score	0.09	0.533	0.10	0.454
	T25FW seconds	0.02	0.890	0.11	0.450
	9HPT seconds	0.25	0.071	0.19	0.178
	SDMT correct answers	−0.52	**<0.001**	−0.08	0.575
	PASAT correct answers	−0.22	0.113	0.03	0.842
	WBV mL	−0.38	**0.005**	0.04	0.751
	CGMV mL	−0.28	**0.038**	0.03	0.814
	DGMV mL	−0.30	**0.028**	0.06	0.691
	T2LV mL	0.46	**<0.001**	0.07	0.614
NfL pg/mL	EDSS score	0.03	0.838	0.10	0.468
	T25FW seconds	−0.02	0.861	0.05	0.745
	9HPT seconds	0.33	**0.015**	**0.39**	**0.004**
	SDMT correct answers	−0.42	**0.002**	−0.21	0.128
	PASAT correct answers	−0.28	**0.040**	−0.17	0.230
	WBV mL	−0.30	**0.030**	−0.00	0.952
	CGMV mL	−0.25	0.066	0.07	0.634
	DGMV mL	−0.29	**0.033**	−0.09	0.541
	T2LV mL	0.38	**0.005**	**0.28**	**0.041**

9HPT: nine-hole peg test; CGMV: cortical grey matter volume; CSF: cerebrospinal fluid; DGMV: deep grey matter volume; EDSS: Expanded Disability Status Scale; NfH: neurofilament heavy; NfL: neurofilament light; PASAT: paced auditory serial addition test; SDMT: symbol digit modalities test; T2LV: T2 lesion volume; T25FW: timed 25-foot walk; WBV: whole brain volume.

Spearman rank’s partial correlations adjusted for age. The number of subjects in all analyses was 54.

Values reported in bold represent statistically significant results (p<0.05).

Higher CSF NfH and NfL were associated with smaller MRI-derived whole brain volume (*r* = −0.38, *p* = 0.005 and *r* = −0.30, *p* = 0.030, respectively), smaller deep grey matter volumes (*r* = −0.30, *p* = 0.028 and *r* = −0.29, *p* = 0.033, respectively) and larger lesion load (*r* = 0.46, *p* < 0.001 and *r* = 0.38, *p* = 0.005, respectively). However, only CSF NfH was correlated with cortical grey matter volume (*r* = −0.28, *p* = 0.038) (Table 2).

At baseline, serum neurofilaments (light and heavy chain) did not generally correlate with any clinical or MRI measure, except for serum NfL, which was a predictor of hand function and lesion volume (Table 2).

### Correlations between neurofilaments at different time points and EDSS and PBVC at 96 weeks

Baseline NfH and NfL in CSF did not correlate with future EDSS at 96 weeks (*r* = 0.27, *p* = 0.061 and *r* = 0.15, *p* = 0.296, respectively). However, CSF NfH at week 48 was associated with EDSS at 96 weeks (*r* = 0.42, *p* = 0.011). Also, CSF NfH at week 96 was significantly associated with EDSS at the same time point (*r* = 0.35, *p* = 0.046) (Table 3).

**Table 3.** Correlation coefficients between CSF neurofilament levels, EDSS score and PBVC at 96 weeks.

	CSF NfH			CSF NfL		
	Baseline (*n* = 50)	48 weeks (*n* = 37)	96 weeks (*n* = 34)	Baseline (*n* = 50)	48 weeks (*n* = 37)	96 weeks (*n* = 34)
EDSS at 96 weeks	*r* = 0.27 *p* = 0.061	*r* **=** **0.42** *p* = 0.011	*r* = 0.35 *p* **=** **0.046**	*r* = 0.15 *p* = 0.296	*r* = 0.27 *p* = 0.108	*r* = 0.22 *p* = 0.214
	Baseline (*n* = 48)	48 weeks (*n* = 35)	96 weeks (*n* = 32)	Baseline (*n* = 48)	48 weeks (*n* = 35)	96 weeks (*n* = 32)
PBVC at 96 weeks	*r* = −0.25 *p* = 0.092	*r* = −0.06 *p* = 0.752	*r* = 0.04 *p* = 0.841	*r* **=** **−0.33** *p* = **0.022**	*r* = −0.26 *p* = 0.144	*r* = −0.03 *p* = 0.863
	Serum NfH			Serum NfL		
	Baseline (*n* = 50)	48 weeks (*n* = 36)	96 weeks (*n* = 33)	Baseline (*n* = 50)	48 weeks (*n* = 36)	96 weeks (*n* = 33)
EDSS at 96 weeks	*r* = 0.14 *p* = 0.334	*r* = 0.11 *p* = 0.509	*r* = 0.14 *p* = 0.450	*r* = 0.12 *p* = 0.409	*r* = −0.03 *p* = 0.853	*r* = 0.21 *p* = 0.243
	Baseline (*n* = 48)	48 weeks (*n* = 34)	96 weeks (*n* = 31)	Baseline (*n* = 48)	48 weeks (*n* = 34)	96 weeks (*n* = 31)
PBVC at 96 weeks	*r* = −0.04 *p* = 0.794	*r* = 0.10 *p* = 0.559	*r* = 0.20 *p* = 0.299	*r* = −0.27 *p* = 0.061	*r* = −0.35 *p* **=** **0.047**	*r* = 0.02 *p* = 0.913

CSF: cerebrospinal fluid; EDSS: Expanded Disability Status Scale; NfH: neurofilament heavy; NfL: neurofilament light; PBVC: percentage brain volume change.

Partial Spearman’s rank correlations adjusted for age. PBVC was measured with the SIENA method; negative correlation coefficients indicate faster brain atrophy.

Values reported in bold represent statistically significant results (p<0.05).

With regard to brain atrophy, the only significant correlation we found was between CSF NfL at baseline and PBVC at 96 weeks (*r* = −0.33, *p* = 0.022), suggesting that larger CSF NfL concentrations at baseline were associated with faster brain atrophy (Table 3).

When looking at serum neurofilaments, we found no statistically significant correlations (Table 3), except for the one between serum NfL at week 48 and PBVC after 96 weeks (*r* = −0.35, *p* = 0.047).

### Longitudinal association between neurofilaments at baseline and other clinical and MRI measures at 48 and 96 weeks

None of the CSF neurofilaments at baseline were associated with future walking ability at week 48 or week 96. CSF neurofilaments at baseline showed significant associations with longitudinal MRI and clinical measures, including hand function and information processing speed, especially in the 96-week follow-up (Table 4). Specifically, CSF NfH and NfL were inversely correlated with information processing speed (e.g. *r* = −0.49, *p* < 0.001 between baseline CSF NfH and 96-week SDMT and *r* = −0.37, *p* = 0.010 between baseline CSF NfL and 96-week SDMT). Still, only baseline CSF NfH was a predictor of worse information processing speed at 48 weeks (*r* = −0.45, *p* = 0.001 for SDMT and *r* = −0.42, *p* = 0.003 for PASAT) (Table 4). The strength of the correlations between baseline CSF neurofilaments and 96-week cognitive tests was higher for NfH than NfL (*r* = −0.49 vs. *r* = −0.37 for SDMT and *r* = −0.45 vs. *r* = −0.33 for PASAT). Both baseline CSF NfH and NfL were inversely correlated with 9HPT at 96 weeks (*r* = 0.35, *p* = 0.016 and *r* = 0.35, *p* = 0.015, respectively), but only baseline CSF NfL was a predictor of worse hand dexterity at 48 weeks (*r* = 0.29, *p* = 0.042) (Table 4).

**Table 4.** Associations between baseline neurofilaments and other clinical and MRI variables at follow-up.

Neurofilaments at baseline		CSF		SERUM	
	Clinical variable at 48 weeks	Corr. coeff.	*p*	Corr. coeff.	*p*
NfH pg/mL	T25FW seconds	0.20	0.168	0.09	0.528
	9HPT seconds	0.23	0.112	0.10	0.480
	SDMT correct answers	−0.45	**0.001**	−0.08	0.563
	PASAT correct answers	−0.42	**0.003**	0.01	0.926
NfL pg/mL	T25FW seconds	0.10	0.498	0.09	0.556
	9HPT seconds	0.29	**0.042**	0.34	**0.016**
	SDMT correct answers	−0.26	0.067	−0.14	0.338
	PASAT correct answers	−0.27	0.055	−0.24	0.098
	Clinical variable at 96 weeks	Corr. coeff.	*p*	Corr. coeff.	*p*
NfH pg/mL	T25FW seconds	0.26	0.072	0.14	0.325
	9HPT seconds	0.35	**0.016**	0.22	0.137
	SDMT correct answers	−0.49	**<** **0.001**	−0.11	0.461
	PASAT correct answers	−0.45	**0.001**	−0.14	0.333
NfL pg/mL	T25FW seconds	0.19	0.189	0.12	0.434
	9HPT seconds	0.35	**0.015**	0.40	**0.004**
	SDMT correct answers	−0.37	**0.010**	−0.20	0.183
	PASAT correct answers	−0.33	**0.023**	−0.42	**0.003**
	MRI variable at 96 weeks	Corr. coeff.	*p*	Corr. coeff.	*p*
NfH pg/mL	WBV mL	−0.44	**0.002**	−0.03	0.821
	CGMV mL	−0.34	**0.021**	−0.08	0.590
	DGMV mL	−0.34	**0.020**	−0.01	0.934
	T2LV mL	0.43	**0.003**	0.14	0.360
NfL pg/mL	WBV mL	−0.37	**0.010**	−0.12	0.436
	CGMV mL	−0.32	**0.027**	−0.02	0.884
	DGMV mL	−0.35	**0.016**	−0.20	0.171
	T2LV mL	0.42	**0.004**	0.31	**0.032**

9HPT: nine-hole peg test; CGMV: cortical grey matter volume; CSF: cerebrospinal fluid; DGMV: deep grey matter volume; EDSS: Expanded Disability Status Scale; NfH: neurofilament heavy; NfL: neurofilament light; PASAT: paced auditory serial addition test; SDMT: symbol digit modalities test; T2LV: T2 lesion volume; T25FW: timed 25-foot walk; WBV: whole brain volume.

Partial Spearman’s rank correlations adjusted for age. The number of subjects included in the analyses was as follows: at week 48, clinical data (T25FW, 9HPT, SDMT, PASAT) available for *n* = 50 (*n* = 4 clinical assessments missing) and at week 96, SDMT and MRI available for *n* = 48; T25FW, 9HPT and PASAT available for *n* = 49.

Values reported in bold indicate statistically significant results (p<0.05).

Both baseline CSF NfH and NfL were similarly correlated with brain and lesion volume measures at 96 weeks (all the correlations are reported in Table 4).

Overall, serum neurofilaments were not associated with clinical and MRI measures at follow-up, except serum NfL at baseline, which was the only predictor of worse hand performance (*r* = 0.40, *p* = 0.004), worse performance on PASAT (*r* = −0.42, *p* = 0.003) and larger T2 lesion load (*r* = 0.31, *p* = 0.032) at 96 weeks (Table 4).

## Discussion

Our analysis suggests that for small sample-sized phase 2 trials in SPMS, CSF neurofilaments are more informative than serum neurofilaments as outcome measures of clinical and MRI worsening over 96 weeks. Our study is novel as we looked at neurofilament heavy chain in a large group of people with SPMS using a contemporary immunoassay (Simoa). The aim of our study was to look at the specific associations between CSF NfH with clinical and MRI measures of disability, expecting that these would be better predictors than CSF NfL over time. This hypothesis was based on the biological rationale that in progressive MS, there is less disease activity and more axonal loss. None of the measured neurofilaments showed consistent associations with EDSS and walking ability. Nevertheless, we found that both baseline CSF neurofilaments (heavy and light chains) were similar predictors of hand dysfunction, information processing speed worsening, and brain volume measures after 96 weeks. However, our data reveal potentially distinct correlation patterns between CSF NfH, CSF NfL, and various clinical and MRI variables, suggesting that these biomarkers may serve complementary roles in reflecting different aspects of neurodegeneration and disease progression. NfL was more predictive of global brain atrophy and hand dexterity over time, while NfH was associated with more severe cognitive deficit and cortical volume loss. A possible interpretation of these findings is that CSF NfH might be more sensitive to widespread, chronic axonal damage shown by the relationship with cortical atrophy at baseline, which could also explain its association with cognitive deficit in SDMT at all time points. CSF NfL, instead, may be more sensitive to detecting early signs of progressive atrophy, as shown by the statistically significant association with brain atrophy after 96 weeks. As secondary outcomes, we looked at serum neurofilaments, finding that only NfL had significant and consistent correlations with hand dexterity, PASAT and lesion volume across the study. A further point that emerged from our study was that CSF sampling can be embedded into trial designs in progressive MS and provide useful information even when the sample size is small.

Studies in relapsing MS have shown that CSF NfL levels are increased during relapses and are positively associated with disability and MRI lesion load, particularly gadolinium enhancement.^15,25^ However, in a cohort like the one we recruited in the MS-SMART trial, where relapses occurred in a small proportion of patients (about 10%) before and during the study, and with a median number of T2 new/enlarged lesions of 0 (interquartile range (IQR) = 0–6) over 96 weeks,^20,22^ the likelihood of observing a change in NfL in relation to disease activity is expected to be low. NfH, therefore, might be a better predictor of disease worsening, as suggested by previous studies where NfH seemed to increase during irreversible late-stage axonal degeneration such as progressive stages of the disease^14,19^ However, we did not find consistent associations between baseline CSF NfH or NfL and EDSS. This would be in contrast with the study from Teunissen et al., where they found that both NfH and NfL were correlated with EDSS. The explanation of these differences likely relies on the differences in the populations included in the two studies. Teunissen and colleagues measured correlations between a larger group of people at different MS stages (relapsing-remitting multiple sclerosis (RRMS) = 42, SPMS = 28, primary-progressive multiple sclerosis (PPMS) = 6, clinically isolated syndrome (CIS) = 38), which is more than double compared to our sample of 54 subjects with SPMS. However, it must be noted that EDSS is not a measure sensitive to subtle changes, and it is mostly affected by mobility. In fact, when we used other clinical measures of disability (information processing speed and hand dexterity), we found significant correlations between baseline CSF NfH and NfL and disability. Notably, while no associations were observed cross-sectionally at baseline, CSF NfH demonstrated positive correlations with EDSS at specific time points. For instance, 48-week CSF NfH correlated with EDSS at 96 weeks (*r* = 0.42, *p* = 0.011) and 96-week CSF NfH correlated with EDSS at the same time point (*r* = 0.35, *p* = 0.046).

Studies examining the relationship between neurofilaments and clinical measures of disability have led to inconsistent results.^23^ For instance, Petzold et al.^26^ found no associations between neurofilaments and EDSS at baseline in progressive versus relapsing MS, and similar findings were reported by Gnanapavan et al.^27^ in the phase 2 Lamotrigine study in SPMS.

Serum NfL is a more desirable biomarker than CSF.Being less invasive, serum NlL has gained traction as the technology has improved.^28^ Although NfH might be a better measure than NfL in progressive MS, there is a scarcity of contemporary studies focusing on NfH in this condition. The translation of NfH from CSF to blood has been challenging due to the low sensitivity of the existing assays. Our study is among the few that have attempted to measure NfH in both CSF and blood using the Simoa assay.^24^ Despite using this method, we did not find a correlation between NfH in CSF and serum (Figure 2(d)), which is in keeping with the findings of Eikelenboom et al.,^28^ who applied traditional enzyme-linked immunosorbent assay (ELISA) and electrochemiluminescence (ECL) assays.^26^

In a small study of 39 MS patients (including seven with progressive MS), Kalatha et al.^29^ found that elevated CSF NfL correlated with cognitive dysfunction in the progressive MS group only. Similarly, in the phase 2 Lamotrigine trial investigating neuroprotection in SPMS, higher CSF NfH concentrations were associated with lower PASAT scores.^27^ Importantly, cognitive impairment in MS is primarily driven by grey matter damage.^30^ In our study, higher NfL and NfH were variably correlated with lower SDMT, PASAT, and whole and grey matter volumes, suggesting that CSF neurofilaments reflect brain volume loss and reduced information processing speed, which are linked to the underlying neurodegenerative process. We did not find any correlation between serum neurofilaments with brain whole or grey matter volumes and SDMT. However, serum neurofilaments correlated with better hand function and larger lesion volume.

The search for treatments to prevent disability progression in MS remains a major unmet need.^31^ So far, only a few trials in progressive MS have included neurofilaments as markers of disease worsening and treatment response, and all these studies have focused on NfL.^6,12,27,32^ For example, the SPRINT-MS study,^32^ which demonstrated that ibudilast could reduce brain atrophy in progressive MS, found no difference in either serum or CSF NfL between treatment arms, possibly due to the limited anti-inflammatory effects of ibudilast. However, they did not measure CSF NfH.

Measuring CSF biomarkers in a clinical trial setting presents challenges, as we experienced during the recruitment of the MS-SMART trial. In this trial, only 55 out of 176 randomized subjects (31%) participated in the optional CSF substudy, and only two-thirds (*n* = 35) completed the CSF follow-up. It could be argued that studies with primary CSF arms, such as the Ocrelizumab Biomarker Outcome Evaluation (OBOE) study with ocrelizumab,^33^ might have achieved better retention rates. Nevertheless, the overall small sample size and the low retention rate (around 60%) are limitations of our study. Despite these challenges, we found that CSF neurofilaments were the only biomarkers that consistently showed associations with future disability and MRI-derived atrophy measures. Therefore, CSF studies using neurofilaments should be considered for small-sized proof-of-concept clinical trials or phase 2 studies. However, serum NfL was consistently associated with hand function and lesion volume, and this finding may have implications in small-sized serum neurofilament studies.

Another limitation of our study is that we did not control for comorbidities in our statistical models. It is known that hypertension and hypercholesterolemia can induce brain damage and have been related to increased NfL levels.

Due to the exploratory nature of this study, we did not adjust for multiple comparisons, and results should be interpreted cautiously.^34^

In conclusion, despite the growing evidence supporting NfL as a blood marker of inflammation in relapsing MS, we still lack a reliable biomarker for evaluating central nervous system injury in progressive MS. Our findings suggest that NfH, when measured in CSF, might have a relevant role as a biomarker of disease worsening in SPMS and be complementary to CSF NfL to reflect different disease worsening patterns. Serum NfL was consistently associated with hand function and lesion volume, which should be further studied in larger serum neurofilament studies. We recommend considering CSF neurofilaments as biomarkers when planning small-sized studies and measuring both NfL and NfH in larger CSF studies.

## Supplemental Material

sj-docx-1-msj-10.1177_13524585241311212 – Supplemental material for Neurofilament heavy chain in secondary progressive multiple sclerosis Supplemental material, sj-docx-1-msj-10.1177_13524585241311212 for Neurofilament heavy chain in secondary progressive multiple sclerosis by Floriana De Angelis, Francesca Ammoscato, Richard A Parker, Domenico Plantone, Anisha Doshi, Nevin A John, Thomas Williams, Jonathan Stutters, Dave MacManus, Klaus Schmierer, Frederik Barkhof, Christopher J Weir, Gavin Giovannoni, Jeremy Chataway and Sharmilee Gnanapavan in Multiple Sclerosis Journal
