# Supplementary material for: Neurofilament heavy chain in secondary progressive multiple sclerosis
Source: Mult Scler. 2025 Jan 22;31(3):303–13. doi: 10.1177/13524585241311212 (PMC11907725; doi:10.1177/13524585241311212)
Supplement: sj-docx-1-msj-10.1177_13524585241311212 – Supplemental material for Neurofilament heavy chain in secondary progressive multiple sclerosis [file sj-docx-1-msj-10.1177_13524585241311212.docx]

**Supplementary material**

**Supplementary table 1. Comparison between baseline characteristics of the cohort recruited in the CSF sub-study and overall cohort recruited at UCL for the MS-SMART trial**

|  | **CSF Sub-study**  **Cohort**  **n=54** | **MS-SMART Trial**  **UCL Cohort**  **N= 176** | **P value** |
| --- | --- | --- | --- |
| **Age, years** | 53.70 (8.16) | 54 (7.2) | 0.798 |
| **Female sex,** % | 31 (57.4) | 122 (69) | - |
| **Disease-duration,** years | 22.05 (9.00) | 22.1 (9.2) | 0.925 |
| **Progression duration,** years | 8.04 (6.07) | 8.6 (5.9) | 0.542 |
| **EDSS,** score | 6.0 (5.625-6.5) | 6.0 (5.5 - 6.5) | 0.604 |
| **9HPT**, sec | 40.67 (53.81) | 49.8 (76.3) | 0.324 |
| **T25FW,** sec | 17.49 (27.00) | 21.5 (32.6) | 0.361 |
| **PASAT,** no. of correct answers | 45.5 (34.25-52.75) | 41 (32.5 – 52) | 0.355 |
| **SDMT,** no. of correct answers | 47.5 (40.25-51) | 49 (39-53) | 0.370 |
| **WBV,** ml | 1408.04 (87.92) | 1412.1 (89.2) | 0.804 |
| **DGMV,** ml | 45.10 (4.55) | 45.4 (4.4) | 0.722 |
| **CGMV,** ml | 786.00 (48.81) | 790.1 (46.7) | 0.607 |
| **T2LV,** ml | 14.13 (11.46) | 12.5 (10.2) | 0.405 |

9HPT: none hole peg test. CGMV: cortical grey matter volume. CSF: cerebrospinal fluid. DGMV: deep grey matter volume. EDSS: expanded disability status scale. NfH: neurofilament heavy. NfL: neurofilament light. PASAT: paced auditory serial addition test. SDMT: symbol digit modalities test. T2LV: T2 lesion volume. T25FW: timed 25-foot walk. WBV: whole brain volume.

**Supplementary Table 2. Extreme outliers**

| **Subject** | **Neurofilament variable** | **value** |
| --- | --- | --- |
| ##21 | Serum NfL | 68.07 pg/ml |
| ##22 | Serum NfH | 359.55 pg/ml |
| ##52 | CSF NfL | 2594.74 pg/ml |

Trial subjects IDs have been further pseudoanonymised.

Abbreviations

CSF: cerebrospinal fluid. NfH: neurofilament heavy (chain). NfL: neurofilament light (chain).

**Supplementary Table 3. Baseline characteristics after removing extreme outliers.**

|  | **All**  **n=51** |
| --- | --- |
| **Age, years** | 53.7 (8.2) |
| **Female sex,** % | 29 (56.9) |
| **Disease-duration,** years | 21.9 (9.2) |
| **Progression duration,** years | 8.0 (6.0) |
| **EDSS,** score | 6.0 (5.5-6.5) |
| **9HPT**, sec | 41.2 (55.3) |
| **T25FW,** sec | 17.8 (27.7) |
| **PASAT,** no. of correct answers | 46 (34.5-53) |
| **SDMT,** no. of correct answers | 48 (41.5-51) |
| **WBV,** ml | 1407.29 (85.03) |
| **DGMV,** ml | 45.14 (4.36) |
| **CGMV,** ml | 785.14 (48.44) |
| **T2LV,** ml | 13.46 (11.40) |
| **CSF NfL,** pg/ml | 942.49 (427.50) |
| **CSF NfH,** pg/ml | 482.30 (139.59) |
| ***Serum NfL,*** pg/ml | *14.65 (4.87)* |
| ***Serum NfH,*** pg/ml | 89.18 (82.54) |

Descriptive statistics are reported as percentage (number), mean (sd) or medians (interquartile range) as appropriate.

Abbreviations: 9HPT: 9-hole peg test. CSF: cerebrospinal fluid. EDSS=expanded disability status scale. WBV= whole brain volume. CGMV= normalized cortical grey matter volume. DGMV= normalized deep grey matter volume. NfL: neurofilament light chain. NfH: neurofilament heavy chain. WBV= normalized whole brain volume. PASAT= paced auditory serial addition test. SDMT=symbol digit modalities test. T25FW: timed 25-foot walk. T2LV= T2 lesion volume.

**Supplementary Table 4 Baseline partial correlations between neurofilaments after removing extreme outliers**

|  | **CSF NfH** | **Serum NfL** | **Serum NfH** |
| --- | --- | --- | --- |
| **CSF NfL** | R= 0.62 p<0.001 | R=0.42 p=0.002 |  |
| **Serum NfL** |  |  | R=0.22 p= 0.129 |
| **CSF NfH** |  |  | R=0.21 p=0.134 |

CSF: cerebrospinal fluid. NfH: neurofilament heavy. NfL: neurofilament light.

**Supplementary Table 5. Partial correlations between NfL and NfH at baseline with clinical and MRI variables at baseline adjusted for age after removing extreme outliers.**

| **Predictor** | **Outcome** | **CSF** | | **SERUM** | | |
| --- | --- | --- | --- | --- | --- | --- |
|  |  | **Corr. Coeff.** | **p-value** | **Corr. Coeff.** | **p-value** | |
| **NfH pg/ml** | EDSS score | 0.06 | 0.678 | 0.08 | 0.559 | |
|  | T25FW sec | 0.01 | 0.932 | 0.08 | 0.469 | |
|  | 9HPT sec | 0.24 | 0.099 | 0.19 | 0.191 | |
|  | SDMT correct answers | -0.48 | **<0.001** | -0.02 | 0.888 | |
|  | PASAT correct answers | -0.18 | 0.202 | 0.09 | 0.523 | |
|  | WBV mm^3^ | -0.37 | **0.009** | 0.11 | 0.473 | |
|  | CGMV mm^3^ | -0.31 | **0.027** | 0.14 | 0.334 | |
|  | DGMV mm^3^ | -0.25 | 0.079 | 0.04 | 0.334 | |
|  | T2LV mm^3^ | 0.43 | **0.002** | 0.03 | 0.859 | |
|  |  |  |  |  |  | |
| **NfL pg/ml** | EDSS score | -0.02 | 0.906 | 0.04 | 0.761 | |
|  | T25FW sec | -0.05 | 0.739 | 0.02 | 0.906 | |
|  | 9HPT sec | 0.33 | **0.018** | **0.40** | **0.004** |  |
|  | SDMT correct answers | -0.36 | **0.010** | -0.14 | 0.332 | |
|  | PASAT correct answers | -0.26 | 0.066 | -0.14 | 0.321 | |
|  | WBV mm^3^ | -0.28 | **0.045** | -0.01 | 0.953 | |
|  | CGMV mm^3^ | -0.28 | 0.048 | 0.03 | 0.149 | |
|  | DGMV mm^3^ | -0.25 | 0.080 | -0.05 | 0.846 | |
|  | T2LV mm^3^ | 0.32 | 0.022 | 0.21 | 0.149 | |

Number of subjects included in the analyses: 51.

The results that differ from the main analyses, are highlighted in yellow.

9HPT: 9-hole peg test. CGMV: cortical grey matter volume. CSF: cerebrospinal fluid. DGMV: deep grey matter volume. EDSS: expanded disability status scale. NfH: neurofilament heavy. NfL: neurofilament light. PASAT= paced auditory serial addition test. SDMT: symbol digit modalities test. T2LV: T2 lesion volume. T25FW: timed 25-foot walk. WBV: whole brain volume.

**Supplementary Table 6. Partial correlation coefficients between CSF neurofilament levels, EDSS score and PBVC at 96 weeks adjusted for age after removing extreme outliers**

|  | **logCSF NfH** | | | **logCSF NfL** | | |
| --- | --- | --- | --- | --- | --- | --- |
|  | *Baseline*  *(n=47)* | *48 weeks*  *(n=35)* | *96 weeks*  *(n=33)* | *Baseline*  *(n=47)* | *48 weeks*  *(n=35)* | *96 weeks*  *(n=33)* |
| **EDSS at 96 weeks** | r= 0.27  p= 0.070 | r= 0.38  p= 0.027 | **r= 0.39**  **p= 0.028** | r= 0.13  p= 0.374 | r= -0.21  p= 0.228 | r= 0.24  p= 0.189 |
|  |  |  |  |  |  |  |
|  | *Baseline*  *(n=45)* | *48 weeks*  *(n=33)* | *96 weeks*  *(n=31)* | *Baseline*  *(n=45)* | *48 weeks*  *(n=33)* | *96 weeks*  *(n=31)* |
| **PBVC at 96 weeks** | r= - 0.18  p= 0.235 | **r= 0.36**  **p= 0.045** | r= 0.07  p= 0.697 | r= - 0.29  p= 0.057 | r= - 0.19  p= 0.309 | r= - 0.01  p= 0.957 |
|  | | | | | | |
|  | **logSerum NfH** | | | **logSerum NfL** | | |
|  | *Baseline*  *(n=47)* | *48 weeks*  *(n=35)* | *96 weeks*  *(n=33)* | *Baseline*  *(n=47)* | *48 weeks*  *(n=35)* | *96 weeks*  *(n=34)* |
| **EDSS at 96 weeks** | r= 0.13  p= 0.383 | r= 0.07  p= 0.695 | r= 0.14  p= 0.450 | r= 0.08  p= 0.582 | r= - 0.09  p= 0.596 | r= 0.21  p= 0.243 |
|  |  |  |  |  |  |  |
|  | *Baseline*  *(n=45)* | *48 weeks*  *(n=33)* | *96 weeks*  *(n=31)* | *Baseline*  *(n=45)* | *48 weeks*  *(n=33)* | *96 weeks*  *(n=31)* |
| **PBVC at 96 weeks** | r= 0.03  p= 0.836 | r= 0.09  p= 0.612 | r= 0.20  p= 0.300 | r= -0.23  p= 0.136 | r= - 0.13  p= 0.471 | r= 0.02  p= 0.913 |
|  |  |  |  |  |  |  |

The results that differ from the main analyses, are highlighted in yellow.

PBVC was measured with the SIENA method; negative correlation coefficients indicate faster brain atrophy.

Abbreviations: CSF: cerebrospinal fluid. EDSS: expanded disability status scale. NfH: neurofilament heavy. NfL: neurofilament light. PBVC: percentage brain volume change.

**Supplementary Table 7. Partial correlations between baseline neurofilaments and other clinical and MRI variables at follow-up adjusted for age after removing extreme outliers**

| **Neurofilaments**  **at baseline** |  | **CSF** | | **SERUM** | |
| --- | --- | --- | --- | --- | --- |
|  | **Clinical Variable**  **at 48 weeks** | **Corr. Coeff.** | **p-value** | **Corr. Coeff.** | **p-value** |
| **NfH pg/ml** | T25FW sec | -0.20 | 0.175 | -0.10 | 0.510 |
|  | 9HPT sec | 0.29 | 0.127 | -0.10 | 0.502 |
|  | SDMT correct answers | -0.40 | **0.005** | -0.01 | 0.945 |
|  | PASAT correct answers | -0.41 | **0.005** | 0.09 | 0.554 |
|  |  |  |  |  |  |
| **NfL pg/ml** | T25FW sec | 0.30 | 0.046 | -0.08 | 0.597 |
|  | 9HPT sec | -0.20 | 0.189 | **0.36** | **0.014** |
|  | SDMT correct answers | -0.30 | 0.048 | -0.09 | 0.537 |
|  | PASAT correct answers | -0.29 | 0.047 | **-0.39** | **0.008** |
|  | **Clinical Variable**  **at 96 weeks** | **Corr. Coeff.** | **p-value** | **Corr. Coeff.** | **p-value** |
| **NfH pg/ml** | T25FW sec | - 0.24 | 0.100 | -0.12 | 0.437 |
|  | 9HPT sec | 0.33 | **0.023** | 0.20 | 0.183 |
|  | SDMT correct answers | - 0.43 | **0.003** | -0.04 | 0.812 |
|  | PASAT correct answers | - 0.41 | **0.004** | -0.08 | 0.585 |
|  |  |  |  |  |  |
| **NfL pg/ml** | T25FW sec | -0.17 | 0.269 | -0.09 | 0.560 |
|  | 9HPT sec | 0.35 | **0.017** | 0.41 | **0.005** |
|  | SDMT correct answers | -0.20 | 0.189 | -0.12 | 0.444 |
|  | PASAT correct answers | -0.27 | 0.066 | -0.39 | **0.008** |
|  | **MRI Variable**  **at 96 weeks** | **Corr. Coeff.** | **p-value** | **Corr. Coeff.** | **p-value** |
| **NfH pg/ml** | WBV mm^3^ | - 0.41 | **0.004** | 0.05 | 0.768 |
|  | CGMV mm^3^ | - 0.34 | **0.026** | -0.05 | 0.760 |
|  | DGMV mm^3^ | - 0.28 | 0.068 | -0.08 | 0.586 |
|  | T2LV mm^3^ | 0.40 | **0.008** | 0.09 | 0.565 |
| **NfL pg/ml** | WBV mm^3^ | -0.34 | **0.022** | -0.11 | 0.492 |
|  | CGMV mm^3^ | -0.33 | **0.028** | -0.04 | 0.778 |
|  | DGMV mm^3^ | -0.28 | 0.066 | -0.18 | 0.250 |
|  | T2LV mm^3^ | 0.36 | **0.017** | 0.23 | **0.126** |

Data available at follow up:

T25FW, 9HPT, PASAT SDMT at 48 weeks: n=47

T25FW, 9HPT, PASAT at 96 weeks: n=47

SDMT at 96 weeks: n= 46

MRI at 96 weeks: n=45

The results that differ from the main analyses, are highlighted in yellow.

9HPT: none hole peg test. CGMV: cortical grey matter volume. CSF: cerebrospinal fluid. DGMV: deep grey matter volume. EDSS: expanded disability status scale. NfH: neurofilament heavy. NfL: neurofilament light. PASAT= paced auditory serial addition test. SDMT: symbol digit modalities test. T2LV: T2 lesion volume. T25FW: timed 25-foot walk. WBV: whole brain volume.

**Supplementary table 8. CSF neurofilaments with EDSS and MRI change over 96 weeks.**

| **Subject**  **(n=54)** | **Age** | **Relapses before trial (count)**  **(n= 4/54)** | **CSF NfL bl**  **(n=54)** | **CSF NfL 96 wks**  **(n=33)** | **CSF NfH bl**  **(n=54)** | **CSF NfH 96 wks**  **(n=33)** | **EDSS bl**  **(n=54)** | **EDSS 96wks**  **(n=50)** | **EDSS change** | **Relapses in trial**  **(n=5/50)** | **T2 new enlarging (n=19/49)** |
| --- | --- | --- | --- | --- | --- | --- | --- | --- | --- | --- | --- |
| ###1 | 56.93 | No | 926.11 | 631.09 | 508.65 | 372.67 | 6.5 | 6.5 | Same | 0 | 0 |
| ###2 | 42.04 | No | 1328.33 | 1369.62 | 758.16 | 478.01 | 4 | 6 | Worse | 1 | 6 |
| ###3 | 57.98 | No | 606.58 | NA | 410.93 | NA | 6.5 | 7 | Worse | 0 | 1 |
| ###4 | 59.26 | No | 708.5 | 806.19 | 565.15 | 529.04 | 6 | 6 | Same | 0 | 0 |
| ###5 | 37.58 | No | 714.26 | 956.3 | 388.59 | 456.35 | 6.5 | 6.5 | Same | 1 | 14 |
| ###6 | 51.54 | No | 702.21 | 613.81 | 454.96 | 444.49 | 6 | 6 | Same | 0 | 0 |
| ###7 | 56.16 | No | 505.21 | NA | 385.24 | NA | 5.5 | 5.5 | Same | 0 | 0 |
| ###8 | 64.82 | No | 1922.85 | NA | 626.72 | NA | 6.5 | 6.5 | Same | 0 | 20 |
| ###9 | 63.53 | No | 990.05 | 870.79 | 637.88 | 524.76 | 4 | 5 | Worse | 0 | 1 |
| ##10 | 34.79 | No | 1920.99 | NA | 521.65 | NA | 6 | 4.5 | Better | 0 | 16 |
| ##11 | ***54.51*** | ***No*** | ***674.78*** | ***NA*** | ***609.59*** | ***NA*** | ***6*** | ***NA*** | ***NA*** | ***NA*** | ***NA*** |
| ##12 | 51.82 | Yes (1) | 557.63 | 643.83 | 394.17 | 385.78 | 6.5 | 6.5 | Same | 0 | 12 |
| ##13 | 58.53 | No | 406.23 | 560.12 | 318.95 | 448.88 | 6.5 | 6.5 | Same | 0 | NA |
| ##14 | 59.17 | No | 828.41 | 795.22 | 574.37 | 632.53 | 6.5 | 6.5 | Same | 0 | 0 |
| ##15 | 60.33 | No | 1284.94 | 658.14 | 428.14 | 383.05 | 6 | 6 | Same | 0 | 21 |
| ##16 | 60.97 | No | 646.26 | 632.51 | 307.01 | 350.22 | 6 | 6 | Same | 0 | 0 |
| ##17 | 46.86 | No | 935.42 | 778.09 | 384.91 | 323.8 | 6.5 | 6.5 | Same | 0 | 12 |
| ##18 | 45.74 | No | 1812.22 | 2965.65 | 484.61 | 678.64 | 6.5 | 8 | Worse | 0 | 47 |
| ##19 | 58 | No | 644.87 | 683.55 | 378.08 | 359.4 | 4 | 4 | Same | 0 | 0 |
| ##20 | 39.17 | No | 925.21 | 1167.58 | 377.28 | 442.53 | 4.5 | 5 | Worse | 1 | 14 |
| ##21 | 62.91 | No | 995.71 | NA | 507.63 | NA | 6.5 | 6.5 | Same | 0 | 0 |
| ##22 | 51.33 | No | 2415.36 | NA | 895.5 | NA | 6.5 | 6.5 | Same | 0 | 2 |
| ##23 | 52.56 | No | 1582.03 | 1923.2 | 771.62 | 783.7 | 5.5 | 6 | Worse | 0 | 0 |
| ##24 | 39.43 | No | 583.76 | 555.8 | 321.01 | 343.57 | 6 | 6 | Same | 0 | 6 |
| ##25 | 49.55 | No | 1251.87 | 2387.86 | 349.75 | 523.79 | 6 | 6.5 | Worse | 0 | 2 |
| ##26 | 61.98 | Yes (1) | 724.24 | 697.39 | 429.98 | 496.82 | 4.5 | 3.5 | Better | 0 | 0 |
| ##27 | ***51.61*** | ***No*** | ***1518.85*** | ***NA*** | ***373.62*** | ***NA*** | ***6.5*** | ***NA*** | ***NA*** | ***NA*** | ***NA*** |
| ##28 | 51.81 | No | 1098.71 | 3691.75 | 397 | 2042.87 | 6.5 | 6.5 | Same | 0 | 20 |
| ##29 | ***41.91*** | ***No*** | ***840.7*** | ***NA*** | ***428.79*** | ***NA*** | ***4.5*** | ***NA*** | ***NA*** | ***NA*** | ***NA*** |
| ##30 | 65.03 | No | 1177.51 | 1031.96 | 558.88 | 527.88 | 6.5 | 6.5 | Same | 0 | 1 |
| ##31 | 65.74 | No | 1089.64 | 626.79 | 509.95 | 370.82 | 6 | 6.5 | Worse | 0 | 0 |
| ##32 | 54.91 | Yes (2) | 762.56 | 665.73 | 367.92 | 332.55 | 6 | 6 | Same | 1 | 0 |
| ##33 | 54.02 | No | 689.91 | NA | 429.43 | NA | 6.5 | 7.5 | Worse | 0 | 2 |
| ##34 | 60.33 | No | 799.43 | NA | 614.62 | NA | 6.5 | 6.5 | Same | 1 | 0 |
| ##35 | 64.72 | No | 870.25 | 695.21 | 511.49 | 562.75 | 6.5 | 6.5 | Same | 0 | 0 |
| ##36 | 50.33 | Yes (1) | 1574.95 | NA | 774.17 | NA | 6 | 6 | Same | 0 | 3 |
| ##37 | 60.18 | No | 562.22 | 684.49 | 478 | 586.09 | 6.5 | 6.5 | Same | 0 | 0 |
| ##38 | 47.05 | No | 1119.7 | NA | 629.22 | NA | 6.5 | 6.5 | Same | 0 | 5 |
| ##39 | 45.41 | No | 649.36 | NA | 372.55 | NA | 4 | 3 | Better | 0 | 0 |
| ##40 | 54.57 | No | 1932.01 | NA | 870.39 | NA | 6 | 6.5 | Worse | 0 | 31 |
| ##41 | 49.7 | No | 487.42 | NA | 339.79 | NA | 4 | 5 | Worse | 0 | 0 |
| ##42 | 50.79 | No | 516.95 | 496.91 | 309.15 | 251.17 | 4.5 | 1.5 | Better | 0 | 0 |
| ##43 | 60.25 | No | 798.52 | 739.03 | 524.12 | 256.74 | 6 | 6 | Same | 0 | 0 |
| ##44 | ***55.16*** | ***No*** | ***1312.43*** | ***NA*** | ***805.55*** | ***NA*** | ***4.5*** | ***NA*** | ***NA*** | ***NA*** | ***NA*** |
| ##45 | 61.58 | No | 775.29 | 603.82 | 427.7 | 476.55 | 6 | 5.5 | Better | 0 | 0 |
| ##46 | 59.1 | No | 872.59 | 1110.04 | 587.7 | 545.2 | 6 | 6.5 | Worse | 0 | 0 |
| ##47 | 60.96 | No | 471.41 | 430.13 | 369.65 | 363.47 | 6.5 | 6 | Better | 0 | 0 |
| ##48 | 55.12 | No | 1763.1 | NA | 540.63 | NA | 6 | 5.5 | Better | 0 | 0 |
| ##49 | 44.07 | No | 410.9 | 560.27 | 336.98 | 340.63 | 6 | 6 | Same | 0 | 0 |
| ##50 | 64.2 | No | 754.76 | 881.54 | 365.5 | 416.56 | 4.5 | 4 | Better | 0 | 0 |
| ##51 | 37.83 | No | 638.46 | 828.1 | 457.37 | 382.73 | 6.5 | 6.5 | Same | 0 | 0 |
| ##52 | 46.2 | No | 2594.74 | 978.23 | 577.66 | 589.05 | 6 | 6 | Same | 0 | 4 |
| ##53 | 45.86 | No | 960.03 | NA | 381.14 | NA | 4.5 | 6 | Worse | 0 | 6 |
| ##54 | 62.11 | No | 436.47 | NA | 448.46 | NA | 6.5 | 6.5 | Same | 0 | 0 |

Trial subject IDs have been further pseudoanonymised.

Subjects at baseline: n=51

Subjects at week 96: n=47 (trial withdrawal in red: n=4)

EDSS worse: n= 13 (28%)

EDSS same: n= 26 (55%)

EDSS better: n= 8 (17%)

PBVC missing: n= 2

PBVC worse (negative): 35/45 (78%)

CSF not done at week 96: n= 14 (30%)

Subjects experiencing relapses during the trial: n=5/50 (10%)

T2 new/enlarging missing at 96 weeks: n= 1

T2 new/enlarging median= 0 (IQR 0-6)

Subjects experiencing >1 new/enlarging T2 lesion during the trial: n= 19/49 (39%)

Abbreviations: Bl: baseline. CSF: cerebrospinal fluid. EDSS: expanded disability status scale. NA: not applicable. NfH: neurofilament heavy (chain). NfL: neurofilament light (chain). PBVC: percentage brain volume change. wks: weeks.
